# Characterization of weaning practices in intensive care units in Global South countries: a clinical practice survey

**DOI:** 10.62675/2965-2774.20260112

**Published:** 2026-09-01

**Authors:** Roque Basoalto Escobar, Aline Braz Pereira, Glauco Adrieno Westphal, Madiha Hashmi, Santiago Lucas Napoli, Senthilkumar Rajagopal, Bharath Kumar Tirupakuzhi Vijayaraghavan, Moses Siaw-Frimpong, Cornelius Sendagire, Mohd Shahnaz Hasan, Chun Pan, Jing-Chao Luo, Nicolas Nin, Miguel Ibarra-Estrada, Carlos Alberto Lescano-Alva, Luis Felipe Reyes, Ignacio Granda Luna, Diptesh Aryal, Alejandro Bruhn, Gustavo Adolfo Ospina-Tascón, Eduardo Kattan, GS-Wean Investigators, Alexandre Biasi Cavalcanti

**Affiliations:** 1 Departamento de Medicina Intensiva Facultad de Medicina Pontificia Universidad Católica de Chile Santiago Chile Departamento de Medicina Intensiva, Facultad de Medicina, Pontificia Universidad Católica de Chile - Santiago, Chile.; 2 Hospital São José Joinville SC Brazil Hospital São José - Joinville (SC), Brazil.; 3 Intensive Care Unit Centro Hospitalar Unimed Joinville SC Brazil Intensive Care Unit, Centro Hospitalar Unimed - Joinville (SC), Brazil.; 4 Department of Critical Care Medicine Ziauddin University Karachi Pakistan Department of Critical Care Medicine, Ziauddin University - Karachi, Pakistan.; 5 Hospital General de Agudos “Dr. Juan A. Fernández” Buenos Aires Argentina Hospital General de Agudos “Dr. Juan A. Fernández” - Buenos Aires, Argentina.; 6 Department of Critical Care Medicine Apollo Hospitals Chennai India Department of Critical Care Medicine, Apollo Hospitals - Chennai, India.; 7 Directorate of Anaesthesia and Intensive Care Komfo Anokye Teaching Hospital Kumasi Ghana Directorate of Anaesthesia and Intensive Care, Komfo Anokye Teaching Hospital - Kumasi, Ghana.; 8 Department of Anesthesia and Critical Care Makerere University College of Health Sciences Kampala Uganda Department of Anesthesia and Critical Care, Makerere University College of Health Sciences - Kampala, Uganda.; 9 Department of Anaesthesiology Faculty of Medicine Universiti Malaya Kuala Lumpur Malaysia Department of Anaesthesiology, Faculty of Medicine, Universiti Malaya - Kuala Lumpur, Malaysia.; 10 Department of Critical Care Medicine Health Management Center University of Electronic Science and Technology of China Chengdu China Department of Critical Care Medicine, Health Management Center, University of Electronic Science and Technology of China, Sichuan Provincial People’s Hospital - Chengdu, China.; 11 Department of Critical Care Medicine Sichuan Academy of Medical Sciences and Sichuan Provincial People’s Hospital Chengdu China Department of Critical Care Medicine, Sichuan Academy of Medical Sciences and Sichuan Provincial People’s Hospital - Chengdu, China.; 12 Unidad de Cuidados Intensivos Hospital Español “Juan Jose Crottoggini” Montevideo Uruguay Unidad de Cuidados Intensivos, Hospital Español “Juan Jose Crottoggini” - Montevideo, Uruguay.; 13 Critical Care Unit Hospital Civil Fray Antonio Alcalde Universidad de Guadalajara Jalisco México Critical Care Unit, Hospital Civil Fray Antonio Alcalde, Universidad de Guadalajara - Jalisco, México.; 14 Department of Critical Care Medicine Edgardo Rebagliati Martins National Hospital Lima Peru Department of Critical Care Medicine, Edgardo Rebagliati Martins National Hospital - Lima, Peru.; 15 Unisabana Center for Translational Science School of Medicine Universidad de La Sabana Chía Colombia Unisabana Center for Translational Science, School of Medicine, Universidad de La Sabana - Chía, Colombia.; 16 Intensive Care Unit Hospital General San Francisco Instituto Ecuatoriano de Seguridad Social Quito Ecuador Intensive Care Unit, Hospital General San Francisco, Instituto Ecuatoriano de Seguridad Social - Quito, Ecuador.; 17 Nepal Intensive Care Research Foundation Kathmandu Nepal Nepal Intensive Care Research Foundation - Kathmandu, Nepal.; 18 SENTINET: Surveillance, Epidemiology, and New Technologies for Infectious Emerging Threats Santiago Chile SENTINET: Surveillance, Epidemiology, and New Technologies for Infectious Emerging Threats - Santiago, Chile.; 19 Department of Intensive Care Medicine Fundación Valle del Lili Cali Colombia Department of Intensive Care Medicine, Fundación Valle del Lili - Cali, Colombia.; 20 Research Institute HCor-Hospital do Coração São Paulo SP Brazil Research Institute, HCor-Hospital do Coração - São Paulo (SP), Brazil.

**Keywords:** Respiration, artificial, Ventilator weaning, Spontaneous breathing trial, Airway extubation, Intensive care units, Global South, Surveys and questionnaires

## Abstract

**Objective:**

To characterize current practices and protocols for mechanical ventilation weaning in intensive care units in Global South countries, describing practice variability and identifying opportunities for standardization that may reduce extubation failure.

**Methods:**

We conducted an international, multicenter electronic survey of healthcare professionals working in intensive care units across Global South countries. A structured questionnaire collected data on current weaning practices, including weaning protocols, spontaneous breathing trials, extubation criteria, post-extubation respiratory support, and management of high-risk patients. Descriptive analyses were used to summarize institutional characteristics and clinical practices.

**Results:**

Of 1,098 responses, 738 participants from Global South intensive care units were included (Latin America: 427, Asia: 278, and Africa: 33). Most respondents were physicians working in mixed medical-surgical units, with a median of 16 intensive care unit beds and substantial variability in staffing ratios across regions. Overall, 74% of intensive care units reported a weaning protocol. The spontaneous breathing trial was the most common method to assess extubation readiness, predominantly using pressure support with positive end-expiratory pressure in high-risk patients, although marked regional variation was observed. Return to mechanical ventilation for 1 hour after a successful spontaneous breathing trial was reported in 20% of cases. Post-extubation respiratory support also varied widely, with non-invasive ventilation more frequently used in Asia and Brazil and high-flow nasal cannula in Africa and China.

**Conclusion:**

Weaning practices in Global South intensive care units are highly heterogeneous. Despite widespread use of key evidence-based strategies, variability in spontaneous breathing trial techniques and post-extubation support highlights the need for context-adapted guidelines and targeted research to improve extubation outcomes.

## INTRODUCTION

Approximately 58% of patients admitted to intensive care units (ICUs) require invasive mechanical ventilation (MV).^(1)^ The spontaneous breathing trial (SBT) is routinely used to evaluate a patient’s ability to tolerate the withdrawal of ventilatory support and to determine readiness for extubation.^(2-9)^ Nevertheless, extubation failure requiring reintubation occurs in 5 - 30% of cases and is associated with increased mortality.^(10-16)^ Several strategies have been proposed to mitigate this risk, including systematic screening for weaning readiness, protocolized weaning approaches,^(7,17-20)^ daily sedation interruption, variations in SBT methods and duration, prophylactic use of non-invasive ventilation (NIV) or high-flow nasal cannula (HFNC) after extubation,^(21-27)^ and 1 hour of MV after a successful SBT.^(28,29)^

International studies have described and characterized MV weaning practices across different countries, including the use of weaning protocols, preferred modes of ventilatory support, SBT techniques, and initial strategies for discontinuing invasive ventilation (such as direct extubation, an initial SBT, or primary tracheostomy).^(1,30-33)^ These studies have revealed substantial global heterogeneity in weaning and liberation practices in ICUs, with most available evidence derived from high-income countries. In contrast, data from low- and middle-income countries remain limited. The term “Global South” refers to a group of countries that share specific socioeconomic, political, and structural characteristics, including marked social inequities, higher levels of poverty, limited access to education, and less-resourced health systems. While no single official definition exists, the concept aligns broadly with the World Health Organization’s frameworks for low- and middle-income countries.^(34)^

This study aimed to characterize current practices and protocols for MV weaning in intensive care units in Global South countries, describing practice variability and identifying opportunities for standardization that may reduce extubation failure. By systematically documenting these practices, the study provides a comprehensive overview of existing approaches and highlights opportunities for standardization, particularly regarding strategies to reduce extubation failure rates.

## METHODS

### Study design and ethics approval

This is an international cross-sectional electronic survey targeting healthcare professionals working in ICUs in Global South countries, reported in accordance with the CROSS guideline (A Consensus-Based Checklist for Reporting of Survey Studies) (Table 1S - Supplementary Material).^(35)^ The study was submitted to and approved by the Research Ethics Committee of *Hospital Municipal São José*, Joinville, Brazil (CAAE 85141324.4.0000.5362).

The Medical Research Ethics Committee of Universiti Malaya Medical Center, Kuala Lumpur, Malaysia (MREC: 2025414-14968) also approved the study. In India, approval was obtained from the ethics committee of the coordinating institution (AMH-C-S-062/04-25). Approval was not necessary in other participant countries.

### Questionnaire and variables included in the survey

A structured questionnaire was administered to ICU healthcare professionals (physicians, respiratory therapists, or nurses) to collect information on their current weaning practices, including SBTs, extubation criteria, post-extubation support, and the management of patients at high risk of weaning failure. A patient was considered at high risk for reintubation if they meet one or more of the following criteria: age > 65 years, congestive heart failure as the cause of intubation, moderate or severe chronic obstructive pulmonary disease, Acute Physiology and Chronic Health Evaluation II (APACHE II) > 12 or Simplified Acute Physiology Score III (SAPS 3) > 50 on extubation day, body mass index > 30, presence of 2 or more comorbidities, ineffective cough or excessive airway secretions, failure in more than one SBT, MV for more than 7 days, risk of post extubation laryngeal edema.^(22,24,36)^ The survey also inquired about institutional protocols, staffing ratios, and decision-making processes related to weaning. There was no single primary outcome, as this was an exploratory survey study. The finalized survey was pretested by the authors and a small subset of the target population to identify and resolve potential issues affecting data quality.

The questionnaire was available in Portuguese, English, Spanish, and Chinese (Table 2S - Supplementary Material). No sensitive or personal data were collected. The survey was anonymous, and participants could choose whether to participate. Records of participation in this study were kept confidential and accessed only by individuals directly involved in the research. The questionnaire link was distributed without using contact lists that allow the identification of participants or the visibility of their contact information by third parties. The questionnaire did not collect direct personal information (such as ID numbers, full names, or national identification numbers) or patient data. No incentives were offered to the participants.

### Informed Consent Form

The Informed Consent Form was presented to participants from Brazil before starting the questionnaire on the digital platform. However, in other countries, a text explaining the study and asking whether the participant consent to participate was provided. This allowed participants to receive information about the study, data collection and processing, ethical considerations, details about the research team, contact information, study objectives, potential risks and benefits, and the intended use of the data collected. To proceed to the next stage (eligibility verification), a “lock” mechanism was configured so that only those who explicitly agree to participate can move forward and access the survey questions.

### Sampling and participants

The participants of this international survey were physicians, respiratory therapists, or nurses who reside in a Global South country and work in an ICU. We allowed more than one response from different respondents in the same hospital. We did not exclude any respondents who completed the questionnaire. We used non-probability snowball sampling, initiated through the dissemination of the survey by ICU leaders and researchers from Global South countries, who further distributed it to professionals within their local networks.

### Survey distribution

The survey was carried out over 9 months, from January 2025 to September 2025. We received institutional support from academic research and Global South networks from Latin America, Asia, and Africa. We distributed the survey to healthcare professionals from ICUs in Brazil through the Brazilian Research in Intensive Care Network (BRICNet) and the *Associação de Medicina Intensiva Brasileira* Network (AMIBNet). Additionally, we invited researchers from various ICUs across Latin America via the Latin American Intensive Care Network (LIVEN).

A public survey link or a QR Code was disclosed via e-mail, message, or shared on social media such as Instagram, Facebook, or WhatsApp. The study data were collected and managed using REDCap electronic data capture tools from HCor, São Paulo, Brazil.^(37,38)^ The web platform SurveyMonkey® was used to distribute the questionnaire across China and Thailand.^(39)^ The survey was completed anonymously. Participants were instructed to submit only one response per individual. However, no technical measures were implemented to prevent multiple submissions from the same participant. The collected information was automatically entered into an Excel spreadsheet (Microsoft® Excel® for Microsoft 365 MSO [Version 2510 Build 16.0.19328.20190] 64-bit).

### Statistical analysis

Descriptive statistical analyses were used to summarize and interpret the survey data. The distribution of continuous variables was assessed using the Kolmogorov-Smirnov test. Continuous variables with normal distribution were described as mean and standard deviation. All continuous variables in this study had non-normal distributions and were presented as medians, interquartile ranges (IQRs), and 95% confidence intervals (95%CIs).^(40,41)^ Categorical variables were reported as absolute counts, denominators, and percentages, with 95%CIs for proportions calculated using the Wilson score method.

Participants who accessed the survey but did not provide any responses were considered unit nonrespondents. As the mechanism underlying their nonresponse may be related to unobserved factors (e.g., lack of interest, time constraints), the missing data mechanism was assumed to be missing not at random. Missing responses were excluded from the denominator when calculating proportions.

Data were categorized into predefined macro-zones. This classification extends traditional continental groupings into standardized analytical units. Brazil and China were analyzed separately from Latin America and Asia, respectively, given their markedly higher number of respondents, which could disproportionately influence aggregated regional estimates.

Comparative visual analyses between countries were performed using heatmaps to identify patterns in SBT modalities, post-extubation support strategies, and institutional protocols. The distribution of the most commonly used SBT methods across regions was assessed based on adjusted standardized residuals. Additionally, choropleth world maps were generated to illustrate the geographic distribution of survey responses and to compare the most frequent weaning practices across countries, as shown in figures 1S - 4S (Supplementary Material). Analyses were performed using R software version 4.4.0 (copyright © 2024 The R Foundation for Statistical Computing).

### Sample size calculation

Our goal was to collect approximately 300 responses. There was no formal sample size calculation; however, with a total of 300 responses, we expected to achieve 95%CIs with a maximum width of 11.5%.

## RESULTS

The total number of responses was 1,098. After excluding 360 participants with blank responses, we included 738 participants in the analysis. The global distribution of participants recruited in the study is shown in figure 1 and table 3S (Supplementary Material).

**Figure 1:**
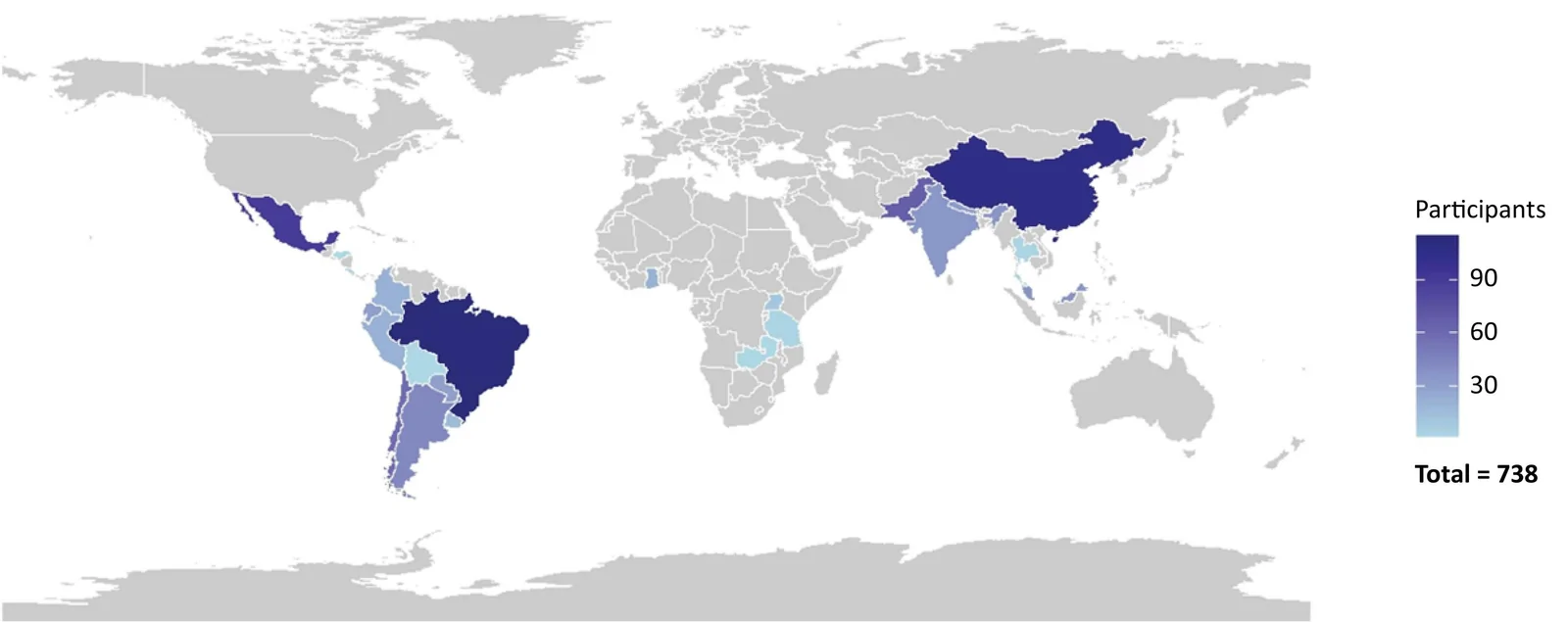
Global distribution of participants recruited in the study. World map illustrates the number of participants recruited per country. A continuous blue color scale was applied, ranging from light blue to dark blue. Lighter shades indicate a smaller number of participants (minimum = 1), while progressively darker shades indicate a greater number of participants, with Brazil showing the highest count (115).

### Hospital characteristics

The most common profession among respondents was physician, followed by respiratory physiotherapists and nurses. Most ICUs were mixed (medical and surgical). The median number of ICU beds was 16 (IQR 10 - 27.5), ranging from 7 (IQR 6 - 12) in Africa to 20 (IRQ 10 - 40) in Brazil and 21 (IQR 13 - 38) in China. There was wide variability in responses regarding the ratio of healthcare professionals (physicians, respiratory physiotherapists, and nurses) to ICU beds across countries (Table 1). Overall, 28% of respondents estimated that 51 - 75% of patients remained on MV for more than 72 hours, with an approximate number of extubations per week of 4.0 (IQR 3 - 8) and 47% of repondents reported a reintubation rate within 7 days among 5 - 10%. Additional characteristics of participating ICUs and respondents are presented in table 1.

**Table 1:** Characteristics of participating intensive care units and respondents

	Overall (n = 738)	Latin America without Brazil (n = 312)	Asia without China (n = 171)	Africa (n = 33)	Brazil (n = 115)	China (n = 107)
Profession						
Physician	503/735 (68.4) (0.64 - 0.71)	200/312 (64.1) (0.58 - 0.69)	102/169 (60.3) (0.52 - 0.67)	23/33 (69.6) (0.52 - 0.82)	80/114 (70.1) (0.61 - 0.77)	98/107 (91.5) (0.84 - 0.95)
Respiratory therapist or physiotherapist	174/735 (23.7) (0.20 - 0.26)	91/312 (29.1) (0.24 - 0.34)	44/169 (26.0) (0.20 - 0.33)	3/33 (9.1) (0.03 - 0.23)	29/114 (25.4) (0.18 - 0.34)	7/107 (6.5) (0.03 - 0.12)
Nurse	58/735 (7.9) (0.06 - 0.10)	21/312 (6.7) (0.04 - 0.10)	23/169 (13.6) (0.09 - 0.19)	7/33 (21.2) (0.10 - 0.37)	5/114 (4.3) (0.01 - 0.09)	2/107 (1.8) (0.005 - 0.06)
ICU beds (median and IQR)	16 (10 - 27.5) (15.0 - 18.0)	14 (10 - 22) (13.0 - 15.0)	20 (11.7 - 27.2) (18.0 - 21.1)	7 (6 - 12) (6.0 - 11.3)	20 (10 - 40) (20.0 - 30.0)	21 (13 - 38) (18.8 - 27.0)
Type of ICU						
Medical	66/734 (8.99) (0.07 - 0.11)	29/309 (9.3) (0.06 - 0.13)	20/170 (11.7) (0.07 - 0.17)	0/33 (0) (0 - 0.10)	12/115 (10.4) (0.06 - 0.17)	5/107 (4.6) (0.02 - 0.10)
Surgical	36/734 (4.90) (0.03 - 0.06)	5/309 (1.6) (0.006 - 0.03)	22/170 (13.0) (0.08 - 0.18)	1/33 (3.0) (0.005 - 0.15)	4/115 (3.4) (0.01 - 0.08)	4/107 (3.7) (0.01 - 0.09)
Mix	632/734 (86.1) (0.83 - 0.88)	275/309 (89.0) (0.85 - 0.92)	128/170 (75.3) (0.68 - 0.81)	32/33 (97.0) (0.84 - 0.99)	99/115 (86.0) (0.78 - 0.91)	98/107 (91.5) (0.84 - 0.95)
Estimated extubation per week (median and IQR)	4.0 (3.0 - 8.0) (4.0 - 5.0)	4.0 (2.0 - 6.0) (3.0 - 4.0)	5 (3.5 - 10) (5.0 - 6.0)	4 (3 - 4.2) (3.0 - 4.0)	3 (2 - 6) (2.1 - 4.0)	8 (3 - 15)) (5.4 - 10.0)
Patients on MV > 72 hours (estimated, %)						
≤ 25%	162/732 (22) (0.19 - 0.25)	61/310 (19.6) (0.15 - 0.24)	66/169 (39) (0.32 - 0.46)	12/33 (36.3) (0.22 - 0.53)	12/113 (10.6) (0.06 - 0.17)	10/107 (9.3) (0.05 - 0.16)
26 - 50%	185/732 (25.3) (0.22 - 0.28)	87/310 (28) (0.23 - 0.33)	35/169 (20.7) (0.15 - 0.27)	6/33 (18.1) (0.08 - 0.34)	24/113 (21.2) (0.14 - 0.29)	33/107 (30.8) (0.22 - 0.40)
51 - 75%	202/732 (27.6) (0.24 - 0.31)	89/310 (28.7) (0.23 - 0.33)	35/169 (20.7) (0.15 - 0.27)	2/33 (6) (0.01 - 0.19)	36/113 (31.8) (0.23 - 0.40)	40/107 (37.3) (0.28 - 0.46)
76 - 100%	101/732 (13.8) (0.11 - 0.16)	54/310 (17.4) (0.13 - 0.22)	8/169 (4.7) (0.02 - 0.09)	1/33 (3) (0.005 - 0.15)	27/113 (23.8) (0.16 - 0.32)	11/107 (10.2) (0.05 - 0.17)
Unknown	82/732 (11.2) (0.09 - 0.13)	19/310 (6) (0.03 - 0.09)	24/169 (14.2) (0.09 - 0.20)	12/33 (36.3) (0.22 - 0.53)	14/113 (12.3) (0.07 - 0.19)	13/107 (12.1) (0.07 - 0.19)
Estimated reintubation rate*						
5 - 10%	342/729 (46.9) (0.43 - 0.50)	131/310 (42.2) (0.36 - 0.47)	92/169 (54.4) (0.46 - 0.61)	13/32 (40.6) (0.25 - 0.57)	43/113 (38.0) (0.29 - 0.47)	63/105 (60.0) (0.50 - 0.68)
10 - 15%	140/729 (19.2) (0.16 - 0.22)	70/310 (22.5) (0.18 - 0.27)	32/169 (19.0) (0.13 - 0.25)	3/32 (9.3) (0.03 - 0.24)	23/113 (20.3) (0.13 - 0.28)	12/105 (11.4) (0.06 - 0.18)
15 - 20%	86/729 (11.8) (0.09 - 0.14)	49/310 (15.8) (0.12 - 0.20)	12/169 (7.1) (0.04 - 0.12)	3/32 (9.3) (0.03 - 0.24)	14/113 (12.3) (0.07 - 0.19)	8/105 (7.6) (0.03 - 0.14)
20 - 25%	34/729 (4.7) (0.03 - 0.06)	18/310 (5.8) (0.03 - 0.08)	4/169 (2.3) (0.009 - 0.05)	1/32 (3.1) (0.005 - 0.15)	9/113 (8.0) (0.04 - 0.14)	2/105 (1.9) (0.005 - 0.06)
25 - 30%	10/729 (1.4) (0.007 - 0.02)	4/310 (1.2) (0.005 - 0.03)	2/169 (1.1) (0.003 - 0.04)	1/32 (3.1) (0.005 - 0.15)	3/113 (2.6) (0.009 - 0.07)	0/105 (0) (0 - 0.03)
> 30%	8/729 (1.1) (0.005 - 0.02)	5/310 (1.6) (0.006 - 0.03)	0/169 (0) (0 - 0.02)	1/32 (3.1) (0.005 - 0.15)	2/113 (1.7) (0.004 - 0.06)	0/105 (0) (0 - 0.03)
Unknown	109/729 (14.9) (0.12 - 0.17)	33/310 (10.6) (0.07 - 0.14)	27/169 (16.0) (0.11 - 0.22)	10/32 (31.3) (0.17 - 0.48)	19/113 (16.8) (0.11 - 0.24)	20/105 (19.0) (0.12 - 0.27)

ICU - intensive care unit; MV – mechanical ventilation. *Reintubation within 7 days post-extubation. Reintubation rate values represent respondents’ self-reported estimates and should be interpreted with caution given potential imprecision. Data are shown as proportions and percentages with 95% confidence intervals, or as medians and interquartile ranges with 95% confidence intervals.

### Weaning practices and spontaneous breathing trial

The strategies and practices for assessing extubation readiness are shown in table 4S (Supplementary Material). According to respondents, 74.2% of ICUs had weaning protocols, ranging from 62.7% in Latin America (without Brazil) to 95.3% in China, most of which were led by physicians, followed by respiratory physiotherapists and an interdisciplinary team.

The SBT was the most reported primary approach for evaluating extubation readiness (50%) across most countries, except in Africa and China, where it was used in combination with a gradual reduction of ventilatory support. This question was not restricted to patients at high risk of reintubation and reflects general practice across all patients. Approximately 80% of respondents reported routinely performing a cuff leak test and administering steroids in patients at risk for post-extubation laryngeal edema.

Spontaneous breathing trial practices are presented in table 2. Spontaneous breathing trials were reported to be used in patients with both low- and high-risk of extubation failure. Twenty percent of respondents reported returning patients to MV for an additional 1 hour after a successful SBT. Among high-risk patients, the most frequently reported SBT modality was pressure support (PS) with positive end-expiratory pressure (PEEP) (54%), followed by the T-piece (21.4%) and PS without PEEP (11%). Pressure support with PEEP was the predominant method in Latin America (without Brazil) (46.3%), Asia (without China) (71.8%), Brazil (44.2%), and China (66.6%), whereas the T-piece was most commonly used in Africa (38%). The most frequently reported SBT technique by country among patients at high risk of extubation failure is shown in figure 1S (Supplementary Material).

**Table 2:** Spontaneous breathing trial practices

	Overall (n = 738)	Latin America without Brazil (n = 312)	Asia without China (n = 171)	Africa (n = 33)	Brazil (n = 115)	China (n = 107)
Patients for whom SBT is performed*						
High risk of weaning failure	507/738 (68.6) (0.65 - 0.72)	216/312 (69.2) (0.63 - 0.74)	110/171 (64.3) (0.56 - 0.71)	17/33 (51.5) (0.35 - 0.67)	83/115 (72.1) (0.63 - 0.79)	81/107 (75.7) (0.66 - 0.82)
Low risk of weaning failure	369/738 (50.0) (0.46 - 0.53)	159/312 (51.0) (0.45 - 0.56)	82/171 (48.0) (0.40 - 0.55)	12/33 (36.3) (0.22 - 0.53)	70/115 (60.8) (0.51 - 0.69)	46/107 (43.0) (0.34 - 0.52)
Other†	75/738 (10.1) (0.08 - 0.12)	34/312 (10.8) (0.07 - 0.14)	15/171 (8.7) (0.05 - 0.13)	5/33 (15.1) (0.06 - 0.30)	12/115 (10.4) (0.06 - 0.17)	9/107 (8.4) (0.04 - 0.15)
Procedure after a successful SBT						
Immediate extubation	546/686 (79.6) (0.76 - 0.82)	241/297 (81.1) (0.76 - 0.85)	126/160 (78.7) (0.71 - 0.84)	25/29 (86.2) (0.69 - 0.94)	83/104 (79.8) (0.71 - 0.86)	71/96 (74.0) (0.64 - 0.81)
MV for 1 hour after successful SBT	140/686 (20.4) (0.17 - 0.23)	56/297 (18.8) (0.14 - 0.23)	34/160 (21.2) (0.15 - 0.28)	4/29 (13.8) (0.05 - 0.30)	21/104 (20.1) (0.13 - 0.28)	25/96 (26.0) (0.18 - 0.35)
High risk of weaning failure SBT modality						
T piece	146/681 (21.4) (0.18 - 0.24)	51/298 (17.1) (0.13 - 0.21)	31/160 (19.3) (0.13 - 0.26)	11/29 (38.0) (0.22 - 0.55)	37/104 (35.5) (0.27 - 0.45)	16/90 (17.7) (0.11 - 0.26)
PS without PEEP	75/681 (11.0) (0.08 - 0.13)	53/298 (17.7) (0.13 - 0.22)	5/160 (3.1) (0.01 - 0.07)	3/29 (10.3) (0.03 - 0.26)	7/104 (6.7) (0.03 - 0.13)	7/90 (7.7) (0.03 - 0.15)
PS with PEEP	367/681 (53.9) (0.50 - 0.57)	138/298 (46.3) (0.40 - 0.51)	115/160 (71.8) (0.64 - 0.78)	8/29 (27.5) (0.14 - 0.45)	46/104 (44.2) (0.35 - 0.53)	60/90 (66.6) (0.56 - 0.75)
Variable	93/681 (13.7) (0.11 - 0.16)	56/298 (18.7) (0.14 - 0.23)	9/160 (5.6) (0.02 - 0.10)	7/29 (24.1) (0.12 - 0.42)	14/104 (13.4) (0.08 - 0.21)	7/90 (7.7) (0.03 - 0.15)
Duration of SBT in minutes						
20	48/687 (7.0) (0.05 - 0.09)	35/298 (11.7) (0.08 - 0.15)	7/160 (4.3) (0.02 - 0.08)	1/29 (3.4) (0.006 - 0.17)	4/104 (3.8) (0.01 - 0.09)	1/96 (1.0) (0.001 - 0.05)
30	272/687 (39.6) (0.36 - 0.43)	130/298 (43.6) (0.38 - 0.49)	59/160 (36.8) (0.29 - 0.44)	4/29 (13.8) (0.05 - 0.30)	43/104 (41.3) (0.32 - 0.50)	36/96 (37.5) (0.28 - 0.47)
60	209/687 (30.4) (0.27 - 0.33)	84/298 (28.1) (0.23 - 0.33)	47/160 (29.3) (0.22 - 0.36)	9/29 (31.0) (0.17 - 0.49)	38/104 (36.5) (0.27 - 0.46)	31/96 (32.2) (0.23 - 0.42)
90	27/687 (3.9) (0.02 - 0.05)	8/298 (2.6) (0.01 - 0.05)	12/160 (7.5) (0.04 - 0.12)	3/29 (10.3) (0.03 - 0.26)	3/104 (2.8) (0.009 - 0.08)	1/96 (1.0) (0.001 - 0.05)
120	118/687 (17.2) (0.14 - 0.20)	38/298 (12.7) (0.09 - 0.17)	32/160 (20.0) (0.14 - 0.26)	12/29 (41.3) (0.25 - 0.59)	16/104 (15.3) (0.09 - 0.23)	20/96 (20.8) (0.13 - 0.30)
Other	12/687 (1.7) (0.01 - 0.03)	3/298 (1.0) (0.003 - 0.02)	3/160 (1.8) (0.006 - 0.05)	0/29 (0) (0 - 0.11)	0/104 (0.0) (0 - 0.03)	6/96 (6.2) (0.02 - 0.12)
Low risk of weaning failure SBT modality						
T piece	179/680 (26.3) (0.23 - 0.29)	82/297 (27.6) (0.22 - 0.32)	31/160 (19.3) (0.13 - 0.26)	15/28 (53.5) (0.35 - 0.70)	29/104 (27.8) (0.20 - 0.37)	22/91 (24.1) (0.16 - 0.33)
PS without PEEP	100/680 (14.7) (0.12 - 0.17)	60/297 (20.2) (0.16 - 0.25)	19/160 (11.8) (0.07 - 0.17)	2/28 (7.1) (0.01 - 0.22)	9/104 (8.6) (0.04 - 0.15)	10/91 (11.0) (0.06 - 0.19)
PS with PEEP	309/680 (45.4) (0.41 - 0.49)	114/297 (38.3) (0.33 - 0.44)	94/160 (58.7) (0.51 - 0.66)	3/28 (10.7) (0.03 - 0.27)	52/104 (50.0) (0.40 - 0.59)	46/91 (50.5) (0.40 - 0.60)
Variable	92/680 (13.5) (0.11 - 0.16)	41/297 (13.8) (0.10 - 0.18)	16/160 (10.0) (0.06 - 0.15)	8/28 (28.5) (0.15 - 0.47)	14/104 (13.4) (0.08 - 0.21)	13/91 (14.2) (0.08 - 0.22)
Duration of SBT in minutes						
20	91/679 (13.4) (0.11 - 0.16)	54/297 (18.1) (0.14 - 0.22)	16/160 (10.0) (0.06 - 0.15)	1/28 (3.5) (0.006 - 0.17)	11/104 (10.5) (0.06 - 0.17)	9/90 (10.0) (0.05 - 0.17)
30	400/679 (58.9) (0.55 - 0.62)	185/297 (62.2) (0.56 - 0.67)	84/160 (52.5) (0.44 - 0.60)	10/28 (35.7) (0.20 - 0.54)	60/104 (57.6) (0.48 - 0.66)	61/90 (67.7) (0.57 - 0.76)
60	131/679 (19.3) (0.16 - 0.22)	41/297 (13.8) (0.10 - 0.18)	48/160 (30.0) (0.23 - 0.37)	7/28 (25.0) (0.12 - 0.43)	22/104 (21.1) (0.14 - 0.29)	13/90 (14.4) (0.08 - 0.23)
90	6/679 (0.9) (0.004 - 0.01)	2/297 (0.6) (0.001 - 0.02)	0/160 (0) (0 - 0.02)	2/28 (7.1) (0.01 - 0.22)	1/104 (0.9) (0.001 - 0.05)	1/90 (1.1) (0.001 - 0.06)
120	46/679 (6.8) (0.05 - 0.08)	13/297 (4.3) (0.02 - 0.07)	10/160 (6.2) (0.03 - 0.11)	8/28 (28.5) (0.15 - 0.47)	9/104 (8.6) (0.04 - 0.15)	6/90 (6.6) (0.03 - 0.13)
Other	5/679 (0.7) (0.003 - 0.01)	2/297 (0.6) (0.001 - 0.02)	2/160 (1.2) (0.003 - 0.04)	0/28 (0) (0 - 0.12)	1/104 (0.9) (0.001 - 0.05)	0/90 (0) (0 - 0.04)

SBT - spontaneous breathing trial; MV - mechanical ventilation; PS - pressure support; PEEP - positive end-expiratory pressure. * One or more alternatives could be selected; † other responses mainly referred to routine application of spontaneous breathing trials across all mechanically ventilated patients, although some respondents specified minimum durations of mechanical ventilation (commonly > 24 hours) or particular clinical exceptions. Data are shown as proportions and percentages, with 95% confidence intervals.

The heatmap in figure 2S (Supplementary Material) illustrates substantial regional variability in preferred SBT techniques. Relative to other regions, T-piece use was higher in Africa and Brazil. Pressure support without PEEP was more commonly used in Latin America (without Brazil) than in other countries, although PS with PEEP was still the most common method in this region. Except for Africa, PS with PEEP was the most frequently used SBT method.

### Post extubation practices

Post-extubation respiratory support strategies are presented in table 3. For patients at high risk of extubation failure, NIV was the most frequently reported modality (54.8%), followed by HFNC (36%). Non-invasive ventilation was the predominant strategy in Asia (without China) (65%) and Brazil (87%), whereas HFNC was most commonly used in Africa (48.4%) and China (59.8%). The most frequently reported post-extubation therapy by country among patients at high risk of extubation failure is shown in figure 3S (Supplementary Material). Overall, 54% (389/719) of respondents reported routinely using active humidification when applying NIV. There was considerable variability in the reported post-extubation support duration.

**Table 3:** Post-extubation respiratory support strategies

	Overall (n = 738)	Latin America without Brazil (n = 312)	Asia without China (n = 171)	Africa (n = 33)	Brazil (n = 115)	China (n = 107)
Routine use of active humidification when applying NIV	388/718 (54.0) (0.50 - 0.57)	159/310 (51.2) (0.45 - 0.56)	89/168 (53.0) (0.45 - 0.60)	19/32 (59.3) (0.42 - 0.74)	29/113 (25.6) (0.18 - 0.34)	92/95 (96.8) (0.91 - 0.98)
High risk of weaning failure						
Type of post-extubation support*						
Standard oxygen therapy	120/738 (16.3) (0.13 - 0.19)	40/312 (12.8) (0.09 - 0.16)	33/171 (19.2) (0.14 - 0.25)	11/33 (33.3) (0.19 - 0.50)	20/115 (17.3) (0.11 - 0.25)	16/107 (15.0) (0.09 - 0.22)
HFNC	265/738 (35.9) (0.32 - 0.39)	116/312 (37.1) (0.32 - 0.42)	58/171 (34.0) (0.27 - 0.41)	16/33 (48.4) (0.32 - 0.64)	11/115 (9.5) (0.05 - 0.16)	64/107 (59.8) (0.50 - 0.68)
NIV	403/738 (54.6) (0.51 - 0.58)	129/312 (41.3) (0.36 - 0.46)	111/171 (65.0) (0.57 - 0.71)	14/33 (42.4) (0.27 - 0.59)	100/115 (87.0) (0.79 - 0.91)	49/107 (45.7) (0.36 - 0.55)
HFNC plus NIV	262/738 (35.5) (0.32 - 0.39)	141/312 (45.1) (0.39 - 0.50)	55/171 (32.1) (0.25 - 0.40)	12/33 (36.3) (0.22 - 0.53)	10/115 (8.6) (0.04 - 0.15)	44/107 (41.1) (0.32 - 0.50)
Duration of support in hours						
12	115/718 (16.0) (0.13 - 0.19)	56/309 (18.1) (0.14 - 0.22)	26/168 (15.4) (0.10 - 0.21)	2/32 (6.2) (0.01 - 0.20)	17/113 (15.0) (0.09 - 0.22)	14/96 (14.5) (0.08 - 0.23)
24	220/718 (30.6) (0.27 - 0.34)	102/309 (33.0) (0.28 - 0.38)	51/168 (30.3) (0.23 - 0.37)	10/32 (31.2) (0.17 - 0.48)	29/113 (25.6) (0.18 - 0.34)	28/96 (29.1) (0.21 - 0.38)
48	96/718 (13.3) (0.11 - 0.16)	43/309 (14.0) (0.10 - 0.18)	19/168 (11.3) (0.07 - 0.16)	1/32 (3.1) (0.005 - 0.15)	27/113 (23.9) (0.16 - 0.32)	6/96 (6.2) (0.02 - 0.12)
No definition	287/718 (40.0) (0.36 - 0.43)	108/309 (35.0) (0.29 - 0.40)	72/168 (42.8) (0.35 - 0.50)	19/32 (59.3) (0.42 - 0.74)	40/113 (35.3) (0.27 - 0.44)	48/96 (50.0) (0.40 - 0.59)
Low risk of weaning failure						
Type of post-extubation support*						
Standard oxygen therapy	615/738 (83.3) (0.80 - 0.85)	273/312 (87.5) (0.83 - 0.90)	144/171 (84.2) (0.78 - 0.88)	24/33 (72.7) (0.55 - 0.84)	103/115 (89.5) (0.82 - 0.93)	71/107 (66.3) (0.56 - 0.74)
HFNC	139/738 (18.8) (0.16 - 0.21)	58/312 (18.5) (0.14 - 0.23)	20/171 (11.6) (0.07 - 0.17)	11/33 (33.3) (0.19 - 0.50)	7/115 (6.0) (0.02 - 0.12)	43/107 (40.1) (0.31 - 0.49)
NIV	60/738 (8.1) (0.06 - 0.10)	10/312 (3.2) (0.01 - 0.05)	15/171 (8.7) (0.05 - 0.13)	3/33 (9.0) (0.03 - 0.23)	19/115 (16.5) (0.10 - 0.24)	13/107 (12.1) (0.07 - 0.19)
HFNC plus NIV	35/738 (4.7) (0.03 - 0.06)	14/312 (4.4) (0.02 - 0.07)	7/171 (4.0) (0.01 - 0.08)	0/33 (0) (0 - 0.10)	5/115 (4.3) (0.01 - 0.09)	9/107 (8.4) (0.04 - 0.15)

NIV - non-invasive ventilation; HFNC - high flow nasal cannula. * One or more alternatives could be selected. Data are shown as proportions and percentages, with 95% confidence intervals.

The heatmap in figure 4S (Supplementary Material) highlights substantial regional variability in post-extubation respiratory support practices. Standard oxygen therapy was used more frequently than expected in Africa. High-flow nasal cannula showed a strong overrepresentation in China. Non-invasive ventilation was more commonly reported in Brazil, whereas its use was lower than expected in Latin America (without Brazil) and China. Combined therapy with NIV and HFNC was markedly overrepresented in Latin America (without Brazil) but was reported less frequently in China and Brazil.

## DISCUSSION

This study provides a multinational description of weaning practices across several regions of the Global South, revealing substantial variation in how key steps of the liberation process are implemented. The findings highlight marked variability in how weaning is conducted, exposing a persistent gap between recommended approaches and routine clinical practice. These results complement prior international studies conducted largely in other regions,^(31,32,42,43)^ while underscoring the challenges that such variability poses for standardization and for developing coherent research and quality-improvement initiatives in diverse ICU settings.

### Respondent profile

The distribution of respondents differed across macrozones. Physicians predominated overall, while physiotherapists or respiratory therapists were more prominent in Brazil and Latin America (excluding Brazil), and nurses constituted the second most common professional group in Africa. Previous international surveys were conducted mainly in North American and European ICUs and described respondent profiles largely dominated by physicians and respiratory therapists.^(31,42)^ In contrast, the Asian survey by Leung et al., which was directed exclusively to physicians, offered limited characterization.^(32)^

### Regional variability in spontaneous breathing trial modality

Variability in SBT practices has been previously reported. A recent survey comparing institutional practice with current clinical practice guidelines in the United States demonstrated only moderate alignment across key components of SBT implementation.^(44)^ Our findings are consistent with these observations and extend them by showing that similar variability is present across multiple regions of the Global South.

One of the most notable findings of our study was the pronounced regional heterogeneity in SBT modality. Previous international surveys have characterized global macrozone variability in SBT practice. Burns et al. described the predominant use of PS with PEEP in North America and Europe, along with persistent use of T-piece trials.^(31)^ Asia (excluding China) showed a strong preference for PS with PEEP, consistent with prior Asian survey data in which 60% of respondents reported using this modality.^(32)^ China, however, displayed a more even distribution of SBT modalities, suggesting the coexistence of traditional and emerging strategies. Latin America (excluding Brazil) reported more frequent use of PS without PEEP, a practice aligned with contemporary evidence.^(45,46)^ Brazil stood out for its dominant use of T-piece trials, a pattern previously noted in large observational studies, but not previously characterized in multinational surveys.^(43,47)^ On the other hand, SBT duration varied less than modality, with most macrozones favoring 30 - 60-minute trials. Longer SBT s in Africa may reflect more conservative decision-making in settings where repeated testing is less feasible or post-extubation support is less frequently used.

### Post-extubation respiratory support

Current evidence and clinical guidelines support the use of prophylactic respiratory support after extubation in high-risk patients, with multiple trials demonstrating that immediate application of HFNC and/or NIV reduces post-extubation respiratory failure and reintubation.^(22,24,48,49)^ Against this well-established evidence base, our survey revealed marked variation in preventive practices across macrozones. China reported predominant use of HFNC, aligning well with current recommendations. Brazil relied more frequently on NIV alone, a pattern consistent with classic evidence.^(47)^ Latin America outside Brazil reported frequent use of alternating HFNC and NIV, representing the approach most closely aligned with contemporary high-risk evidence.^(24,50,51)^ In contrast, Africa continued to rely predominantly on standard oxygen, a modality consistently shown to be inferior to both HFNC and NIV in high-risk populations and no longer recommended.^(48,49)^ The persistent reliance on standard oxygen in Africa likely reflects structural constraints, including limited access to HFNC and NIV devices, consumables, and trained staff, rather than reluctance to adhere to the guidelines. This underscores that translating guideline recommendations into practice in the Global South requires parallel investments in infrastructure and the workforce, not just the dissemination of evidence.

### Short-period mechanical ventilation after spontaneous breathing trial

Immediate extubation after a successful SBT was the predominant strategy across macrozones in our survey (≈80%), with only a minority reporting a 1-hour reconnection period. This pattern likely reflects the inconsistency of the available evidence: Fernandez et al. observed a marked reduction in reintubation with a 1-hour MV reconnection, plausibly through recovery from respiratory muscle fatigue and reversal of derecruitment,^(28)^ whereas Dadam et al. found no overall benefit except in patients ventilated for more than 72 hours.^(29)^ Such discrepancies are partly explained by methodological differences (long, demanding T-piece trials in a high-risk population versus shorter T-piece trials within a rigid extubation protocol). These conditions modulate both the physiologic burden of the SBT and the potential value of a rest period. Notably, both trials relied almost exclusively on T-piece SBT, in contrast to the assisted SBT modalities commonly reported in our survey and supported by current evidence. This mismatch suggests that the physiologic rationale for post-SBT rest may not translate directly to contemporary assisted weaning strategies, which may explain its limited adoption and highlights the need to reassess its relevance in modern practice. Notably, in our survey, we were unable to stratify reconnection practice by risk of reintubation because this question was not asked separately for high- and low-risk patients. This represents a limitation in interpreting our findings, given evidence suggesting that the benefit of reconnection may be greater among patients at higher risk of reintubation.^(29)^

### Limitations

This study has several limitations. First, participation was voluntary, which may introduce selection bias and potentially overrepresent academic or tertiary ICUs. Second, sampling was uneven across countries, limiting the precision of macrozone-level comparisons. Third, all responses were self-reported, making them susceptible to social desirability bias and to clinicians reporting what they believed should occur rather than what actually occurred in clinical practice. Finally, as with all survey-based research, we could not verify practice patterns through direct observation. These limitations are consistent with those described in previous international surveys but should be considered when interpreting our findings.

### Strengths

This study offers several strengths. It provides the largest and most geographically diverse characterization of weaning practices across the Global South, enabling the identification of macrozone-specific patterns not captured in previous surveys. The questionnaire covered key domains, including SBT modality, post-extubation support, and extubation workflow, allowing a granular assessment of practice variability and implementation gaps. Additionally, the inclusion of multidisciplinary ICU clinicians enhances the external validity of the findings and reflects real-world decision-making in contemporary critical care.

## CONCLUSION

This multinational survey reveals substantial, structured heterogeneity in weaning practices across the Global South countries. While several evidence-based strategies are widely implemented, important gaps persist, particularly regarding spontaneous breathing trial modality and standardized post-extubation support. These findings suggest the potential value of context-sensitive guidelines and targeted research to optimize liberation from mechanical ventilation in resource-limited settings.

## SUPPLEMENTARY MATERIAL

SUPPLEMENTARY MATERIAL
