# Supplementary material for: Characterization of weaning practices in intensive care units in Global South countries: a clinical practice survey
Source: Crit Care Sci. 2026 Sep 1;38:e20260112. doi: 10.62675/2965-2774.20260112 (PMC13690035; doi:10.62675/2965-2774.20260112)
Supplement: Supplementary file 1 [file 2965-2774-ccsci-38-e20260112-suppl01.pdf]

*Critical Care Science*
https://doi.org/10.62675/2965-2774.20260112

SUPPLEMENTARY MATERIAL

# Characterization of weaning practices in intensive care units in Global South countries: a clinical practice survey

Roque Basoalto Escobar[1*], Aline Braz Pereira[2*], Glauco Adrieno Westphal[3], Madiha Hashmi[4], Santiago Lucas Napoli[5], Senthilkumar Rajagopal[6], Bharath Kumar Tirupakuzhi Vijayaraghavan[6], Moses Siaw-Frimpong[7], Cornelius Sendagire[8], Mohd Shahnaz Hasan[9], Chun Pan[10], Jing-Chao Luo[11], Nicolas Nin[12], Miguel Ibarra-Estrada[13], Carlos Alberto Lescano-Alva[14], Luis Felipe Reyes[15], Ignacio Granda Luna[16], Diptesh Aryal[17], Alejandro Bruhn[18], Gustavo Adolfo Ospina-Tascón[19], Eduardo Kattan[1], Alexandre Biasi Cavalcanti[20], for the GS-Wean Investigators

**Table 1S -** Checklist for reporting results of internet e-surveys

| Item Category | Checklist Item | Explanation | Page in which item is reported |
|---|---|---|---|
| Title and abstract | 1a | State the word "survey" along with a commonly used term in the title or abstract to introduce the study's design. | 1 |
| | 1b | Provide an informative summary in the abstract, covering background, objectives, methods, findings/results, interpretation/discussion, and conclusions. | 4 |
| Introduction/ background | 2 | Provide background about the rationale of the study, what has been previously done, and why this survey is needed | 5 |
| Purpose/aim | 3 | Identify specific purposes, aims, goals, or objectives of the study. | 5 |
| Methods/ study design | 4 | Specify the study design in the "Methods" section with a commonly used term (e.g., cross-sectional or longitudinal). | 6 |
| Data collection methods | 5a | Describe the questionnaire (e.g., number of sections, number of questions, number and names of instruments used). | 6-7 Suppl material |
| | 5b | Describe all questionnaire instruments that were used in the survey to measure particular concepts. Report target population, reported validity and reliability information, scoring/classification procedure, and reference links (if any). | 6-8 Suppl material |
| | 5c | Provide information on pretesting of the questionnaire, if performed (in the article or in an online supplement). Report the method of pretesting, number of times the questionnaire was pre-tested, number and demographics of participants used for pretesting, and the level of similarity of demographics between pre-testing participants and sample population. | 6-7 |
| | 5d | Questionnaire, if possible, should be fully provided (in the article, or as appendices or as an online supplement). | Suppl material |
| Sample characteristics | 6a | Describe the study population (i.e., background, locations, eligibility criteria for participant inclusion in survey, exclusion criteria). | 7 |
| | 6b | Describe the sampling techniques used (e.g., single stage or multistage sampling, simple random sampling, stratified sampling, cluster sampling, convenience sampling). Specify the locations of sample participants whenever clustered sampling was applied. | 7 |
| | 6c | Provide information on sample size, along with details of sample size calculation. | 8 |
| | 6d | Describe how representative the sample is of the study population (or target population if possible), particularly for population-based surveys. | 8 |
| Survey administration | 7a | Provide information on modes of questionnaire administration, including the type and number of contacts, the location where the survey was conducted (e.g., outpatient room or by use of online tools, such as SurveyMonkey). | 8 |
| | 7b | Provide information on the survey's time frame, such as periods of recruitment, exposure, and follow-up days. | 8 |
| | 7c | Provide information on the entry process:<br>–> For non-web-based surveys, provide approaches to minimize human error in data entry.<br>–> For web-based surveys, provide approaches to prevent "multiple participation" of participants. | - |
| Study preparation | 8 | Describe any preparation process before conducting the survey (e.g., interviewers' training process, advertising the survey). | 6 |

Continue...



This is an open access article under the CC BY license (https://creativecommons.org/licenses/by/4.0/).

...continuation

| | | | |
|---|---|---|---|
| Ethical considerations | 9a | Provide information on ethical approval for the survey if obtained, including informed consent, institutional review board approval, Helsinki declaration, and good clinical practice declaration (as appropriate). | 6 |
| | 9b | Provide information about survey anonymity and confidentiality and describe what mechanisms were used to protect unauthorized access. | 7 |
| Statistical analysis | 10a | Describe statistical methods and analytical approach. Report the statistical software that was used for data analysis. | 8-9 |
| | 10b | Report any modification of variables used in the analysis, along with reference (if available). | 8-9 |
| | 10c | Report details about how missing data was handled. Include rate of missing items, missing data mechanism (i.e., missing completely at random [MCAR], missing at random [MAR], or missing at random [MNAR]), and methods used to deal with missing data (e.g., multiple imputation). | 8-9 |
| | 10d | State how non-response error was addressed. | 8-9 |
| | 10e | For longitudinal surveys, state how loss to follow-up was addressed. | - |
| | 10f | Indicate whether any methods such as weighting of items or propensity scores have been used to adjust for non-representativeness of the sample. | - |
| | 10g | Describe any sensitivity analysis conducted. | - |
| Results/ respondent characteristics | 11a | Report numbers of individuals at each stage of the study. Consider using a flow diagram, if possible. | 9-11 |
| | 11b | Provide reasons for non-participation at each stage, if possible. | - |
| | 11c | Report response rate; present the definition of response rate or the formula used to calculate response rate. | - |
| | 11d | Provide information to define how unique visitors are determined. Report number of unique visitors along with relevant proportions (e.g., view proportion, participation proportion, completion proportion). | - |
| Descriptive results | 12 | Provide characteristics of study participants, as well as information on potential confounders and assessed outcomes. | 9-11<br>20-27<br>Suppl material |
| Main findings | 13a | Give unadjusted estimates and, if applicable, confounder-adjusted estimates along with 95% confidence intervals and p values. | 9-11<br>20-27<br>Suppl material |
| | 13b | For multivariable analysis, provide information on the model building process, model fit statistics, and model assumptions (as appropriate). | - |
| | 13c | Provide details about any sensitivity analysis performed. If there is a considerable amount of missing data, report sensitivity analyses comparing the results of complete cases with those of the imputed dataset (if possible). | - |
| Discussion<br>Limitations | 14 | Discuss the limitations of the study, considering sources of potential biases and imprecision, such as non-representativeness of sample, study design, and important uncontrolled confounders. | 11-14 |
| Interpretations | 15 | Give a cautious overall interpretation of results, based on potential biases and imprecision, and suggest areas for future research. | 11-14 |
| Generalizability | 16 | Discuss the external validity of the results. | 15 |
| Other sections<br>Role of the funding source | 17 | State whether any funding organization has had any roles in the survey's design, implementation, and analysis. | 2-3 |
| Conflict of interest | 18 | Declare any potential conflict of interest. | 3 |
| Acknowledgements | 19 | Provide names of organizations/ persons that are acknowledged along with their contribution to the research. | 15 |

Source: Sharma A, Minh Duc NT, Luu Lam Thang T, Nam NH, Ng SJ, Abbas KS, et al. A Consensus-Based Checklist for Reporting of Survey Studies (CROSS). J Gen Intern Med. 2021;36(10):3179-87.[35]

**Table 2S -** Survey

## QUESTIONNAIRE

- **Do you reside in a Global South country and work in an intensive care unit?**

1. Yes.
2. No.

**Global South Countries:** Low- and middle-income countries predominantly located in the Southern Hemisphere, though some in the Northern Hemisphere are also considered. Examples are listed below. If you are unsure whether your country is part of the Global South, click "yes."

**Examples of Global South Countries/Regions:**

**1. Latin America and the Caribbean** - all countries

**2. Africa** - all countries

**3. Asia (developing regions):**

– India.
– China.
– Indonesia.
– Philippines.
– Vietnam.
– Malaysia.
– Pakistan, among others.

**4. Middle East (some countries, depending on the context):**

– Iran.
– Syria.
– Yemen.
– Oman.

**5. Oceania (some island nations):**

– Papua New Guinea.
– Fiji.

- **What is your region of origin?**

1. Latin America and the Caribbean.
2. Africa.
3. Asia.
4. Oceania.

- **What is your country?**

- **Latin America and the Caribbean**

1. Antigua and Barbuda.
2. Argentina.
3. Bahamas.
4. Barbados.
5. Belize.
6. Bolivia.
7. Brazil.
8. Chile.
9. Colombia.
10. Costa Rica.
11. Cuba.
12. Dominica.
13. Dominican Republic.
14. Ecuador.

15. El Salvador.
16. Grenada.
17. Guatemala.
18. Guyana.
19. Haiti.
20. Honduras.
21. Jamaica.
22. Mexico.
23. Nicaragua.
24. Panama.
25. Paraguay.
26. Peru.
27. Saint Lucia.
28. Saint Kitts and Nevis.
29. Saint Vincent and the Grenadines.
30. Suriname.
31. Trinidad and Tobago.
32. Uruguay.
33. Venezuela.

- **Africa**

1. South Africa.
2. Angola.
3. Algeria.
4. Benin.
5. Botswana.
6. Burkina Faso.
7. Burundi.
8. Cape Verde.
9. Cameroon.
10. Chad.
11. Comoros.
12. Democratic Republic of the Congo.
13. Republic of the Congo.
14. Ivory Coast.
15. Djibouti.
16. Egypt.
17. Eritrea.
18. Eswatini (Swaziland).
19. Ethiopia.
20. Gabon.
21. Gambia.
22. Ghana.
23. Guinea.
24. Guinea-Bissau.
25. Equatorial Guinea.
26. Lesotho.
27. Liberia.
28. Libya.
29. Madagascar.
30. Malawi.
31. Mali.
32. Morocco.
33. Mauritania.
34. Mauritius.
35. Mozambique.
36. Namibia.
37. Niger.
38. Nigeria.
39. Rwanda.
40. São Tomé and Príncipe.
41. Senegal.
42. Seychelles.
43. Sierra Leone.
44. Somalia.
45. Sudan.
46. South Sudan.
47. Tanzania.
48. Togo.
49. Tunisia.
50. Uganda.
51. Zambia.
52. Zimbabwe.

- **Asia**

1. China.
2. Afghanistan.
3. Saudi Arabia.
4. Bangladesh.
5. Bahrain.
6. Bhutan.
7. Brunei.
8. Cambodia.
9. Qatar.
10. United Arab Emirates.
11. Philippines.
12. India.
13. Indonesia.
14. Iran.
15. Iraq.
16. Yemen.
17. Jordan.
18. Kuwait.
19. Laos.
20. Lebanon.
21. Malaysia.
22. Maldives.
23. Myanmar (Burma).

24. Mongolia.
25. Nepal.
26. Oman.
27. Pakistan.
28. Syria.
29. Sri Lanka.
30. Thailand.
31. Timor-Leste.
32. Uzbekistan.
33. Vietnam.

- **Oceania**

1. Fiji.
2. Marshall Islands.
3. Solomon Islands.
4. Kiribati.
5. Micronesia.
6. Nauru.
7. Papua New Guinea.
8. Samoa.
9. Tonga.
10. Tuvalu.
11. Vanuatu.

- **What is your city?**

- **Name of the hospital where you work:**

- **Professional Role:**

1. Physician.
2. Respiratory Physiotherapist.
3. Nurse.

- **Number of ICU beds in your unit:**

- **Type of patients in your ICU:**

1. Medical.
2. Surgical.
3. Both, medical and surgical.

- **Physician-to-patient ratio in your ICU (e.g., if there is 1 doctor for every 8 beds, the answer is 1:8):**

1. 1:2.
2. 1:3.
3. 1:4.
4. 1:5.
5. 1:6.
6. 1:7.
7. 1:8.
8. 1:9.
9. 1:10.
10. 1 physician for more than 10 beds.

- **Nurse-to-patient ratio in your ICU (e.g., if there is 1 nurse for every 2 beds, the answer is 1:2):**

1. 1:2.
2. 1:3.
3. 1:4.
4. 1:5.
5. 1:6.
6. 1:7.
7. 1:8.

8. 1:9.
9. 1:10.
10. 1 nurse for more than 10 beds.

- **Respiratory therapist (or equivalent)-to-patient ratio in your ICU (e.g., if there is 1 respiratory therapist for every 8 beds, the answer is 1:8):**

1. 1:2.
2. 1:3.
3. 1:4.
4. 1:5.
5. 1:6.
6. 1:7.
7. 1:8.
8. 1:9.
9. 1:10.
10. 1 respiratory therapist for more than 10 beds.
11. We do not have a respiratory therapist (or equivalent).

- **In your practice, who usually leads the weaning process (spontaneous breathing trial, extubation, and post-extubation respiratory support)?**

1. Respiratory therapist.
2. Nurse.
3. Physician.
4. Interdisciplinary Team.

- **Describe the approximate number of extubations per week in a typical week at your institution:**

- **What percentage of extubated patients remained on mechanical ventilation for more than 72 hours?**

1. 0 - 25%.
2. 25 - 50%.
3. 50 - 75%.
4. 75 - 100%.
5. I don't have this information.

- **Does your ICU have weaning protocols?**

1. Yes.
2. No.

- **If you use a weaning protocol, which items are included? (Select one or more alternatives):**

1. Checklist to assess eligibility for weaning and spontaneous breathing trials.
2. Spontaneous breathing trials.
3. Evaluation and management of laryngeal edema risk.
4. Cough strength assessment.
5. Use of non-invasive ventilation and/or high-flow nasal cannula after extubation in patients at high risk of extubation failure.
6. Other.

Other (specify):

- **What is the estimated reintubation rate in your ICU (reintubation within 7 days post-extubation)?**

1. 5 - 10%.
2. 10 - 15%.

3. 15 - 20%.
4. 20 - 25%.
5. 25 - 30%.
6. Above 30%.
7. I don't have this information.

• **For the following questions, consider the high-risk criteria for weaning failure. A patient is considered at high risk for reintubation if they meet one or more of the following criteria**:
o Age > 65 years.
o Congestive heart failure as the cause of intubation.
o Moderate or severe chronic obstructive pulmonary disease.
o APACHE II > 12 or SAPS3 > 50 on extubation day.
o Body mass index (BMI) > 30.
o Presence of 2 or more comorbidities.
o Ineffective cough or excessive airway secretions (> 2 aspirations in 8 hours pre-extubation).
o Failure in more than one spontaneous breathing trial.
o Mechanical ventilation for more than 7 days.
o Risk of laryngeal edema (patient with more than 72 hours of mechanical ventilation and at least one of the following criteria: female gender, difficult intubation, traumatic intubation, previous self-extubation).

• **What strategy do you use to assess readiness for extubation?**
1. Spontaneous breathing trial.
2. Gradual reduction of support and extubation without a spontaneous breathing trial.
3. Use both strategies, depending on the patient type.
4. Other.
Other (specify):

• **In which patients do you perform a spontaneous breathing trial (select one or more alternatives)?**
1. Patients at high risk of extubation failure.
2. Patients at low risk of extubation failure.
3. Other.
Other (specify):

• **For high-risk patients, what types of spontaneous breathing trials do you commonly use?**
1. T-piece.
2. Pressure support without PEEP.
3. Pressure support with PEEP.
4. Variable.

• **If you use multiple types of trials, provide an approximate proportion of each (e.g., T-piece: X%; PS with PEEP: Y%; PS without PEEP: Z%). Select more than one alternative:**
1. T-piece: 0 - 25%.
2. T-piece: 25 - 50%.
3. T-piece: 50 - 75%.
4. T-piece: 75 - 100%.
5. PS with PEEP: 0 - 25%.
6. PS with PEEP: 25 - 50%.
7. PS with PEEP: 50 - 75%.
8. PS with PEEP: 75 - 100%.
9. PS without PEEP: 0 - 25%.

10. PS without PEEP: 25 - 50%.
11. PS without PEEP: 50 - 75%.
12. PS without PEEP: 75 - 100%.

- **For high-risk patients, how long do you usually conduct the spontaneous breathing trial?**

1. 20 minutes.
2. 30 minutes.
3. 60 minutes.
4. 90 minutes.
5. 120 minutes.
6. Other.

Other (describe in minutes):

- **For high-risk patients, what post-extubation therapy do you routinely use? (select one or more alternatives)**

1. Conventional oxygen therapy.
2. High-flow nasal cannula (HFNC).
3. Non-invasive ventilation (NIV).
4. NIV alternated with HFNC.

- **Do you routinely use active humidification when applying noninvasive ventilation?**

1. Yes.
2. No.
3. Sometimes.

- **For high-risk patients, what is the minimum duration of post-extubation support?**

1. 12 hours.
2. 24 hours.
3. 48 hours.
4. We do not define a minimum duration.

- **For lo w-risk patients (e.g., those who do not meet the high-risk criteria mentioned above), what types of spontaneous breathing trial do you commonly use?**

1. T-piece.
2. Pressure support without PEEP.
3. Pressure support with PEEP.
4. Variable.

- **If you use multiple types of trials, please specify the proportion of each (e.g., T-piece: X%; PS with PEEP: Y%; PS without PEEP: Z%). Select more than one alternative:**

1. T-piece: 0 - 25%.
2. T-piece: 25 - 50%.
3. T-piece: 50 - 75%.
4. T-piece: 75 - 100%.
5. PS with PEEP: 0 - 25%.
6. PS with PEEP: 25 - 50%.
7. PS with PEEP: 50 - 75%.
8. PS with PEEP: 75 - 100%.
9. PS without PEEP: 0 - 25%.
10. PS without PEEP: 25 - 50%.
11. PS without PEEP: 50 - 75%.
12. PS without PEEP: 75 - 100%.

- **For low-risk patients, how long do you conduct the spontaneous breathing trial?**

1. 20 minutes.
2. 30 minutes.
3. 60 minutes.
4. 90 minutes.
5. 120 minutes.
6. Other.

Other (describe in minutes):

- **For low-risk patients, what type of post-extubation therapy do you routinely use? (select one or more alternatives)**

1. Conventional oxygen therapy.
2. High-flow nasal cannula (HFNC).
3. Non-invasive ventilation (NIV).
4. NIV alternated with HFNC.

- **What is the usual immediate procedure after a successful spontaneous breathing trial?**

1. Extubation immediately after a successful spontaneous breathing trial.
2. Reconnection to mechanical ventilation for 1 hour (with previous ventilatory settings), followed by extubation.

- **Is the cuff leak test performed in your ICU to assess the risk of post-extubation laryngeal edema?**

1. Yes.
2. No.

- **Are corticosteroids commonly used in patients at risk of laryngeal edema to prevent post-extubation complications?**

1. Yes.
2. No.

- **When assessing eligibility for weaning and extubation, do you assess cough strength in all patients? If so, how do you evaluate cough strength?**

1. No, we do not assess cough strength in all patients.
2. Peak cough flow meter.
3. Internal flow meter on the mechanical ventilator.
4. Other.

Other (specify):

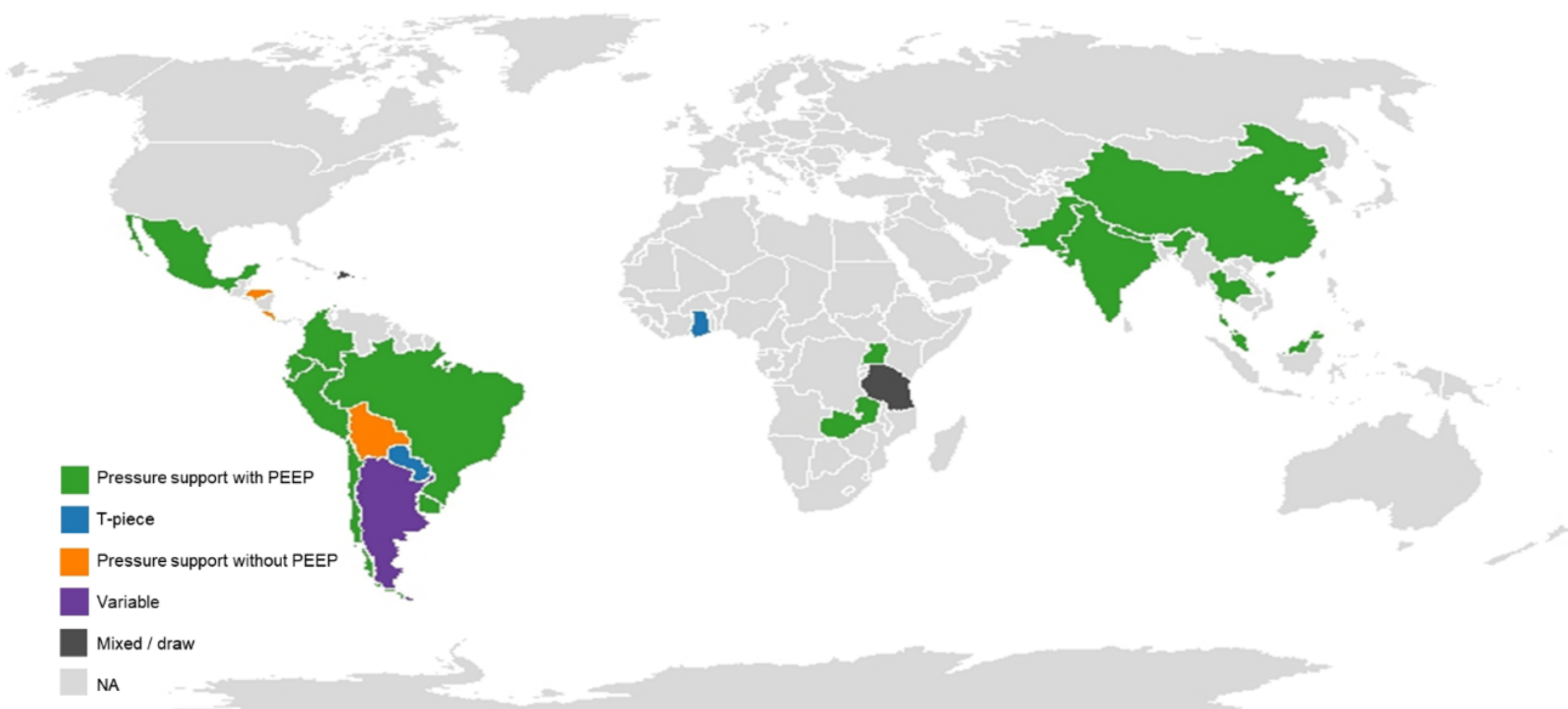


PEEP - positive end-expiratory pressure; NA - not available.

**Figure 1S -** Most frequent spontaneous breathing trial per country in patients with high risk of extubation failure.

World map illustrating the proportion of responses by country: Brazil 46/104 (44%), China 60/90 (66.6%), Argentina 15/42 (36%), Bolivia 1/1 (100%), Chile 29/58 (50%), Colombia 11/21 (52%), Costa Rica 2/2 (100%), Ecuador 11/30 (36.6%), Honduras 1/1 (100%), Mexico 48/89 (54%), Paraguay 15/30 (50%), Peru 11/21 (52%), Uruguay 10/16 (62.5%), Ghana 11/20 (55%), Tanzania 2/3 (66.6%), Uganda 4/9 (44.4%), Zambia 1/1 (100%) India 17/35 (48.5%), Malaysia 33/36 (91.6%), Nepal 27/31 (87%), Pakistan 36/66 (54.5%), Thailand 2/3 (66.6%).

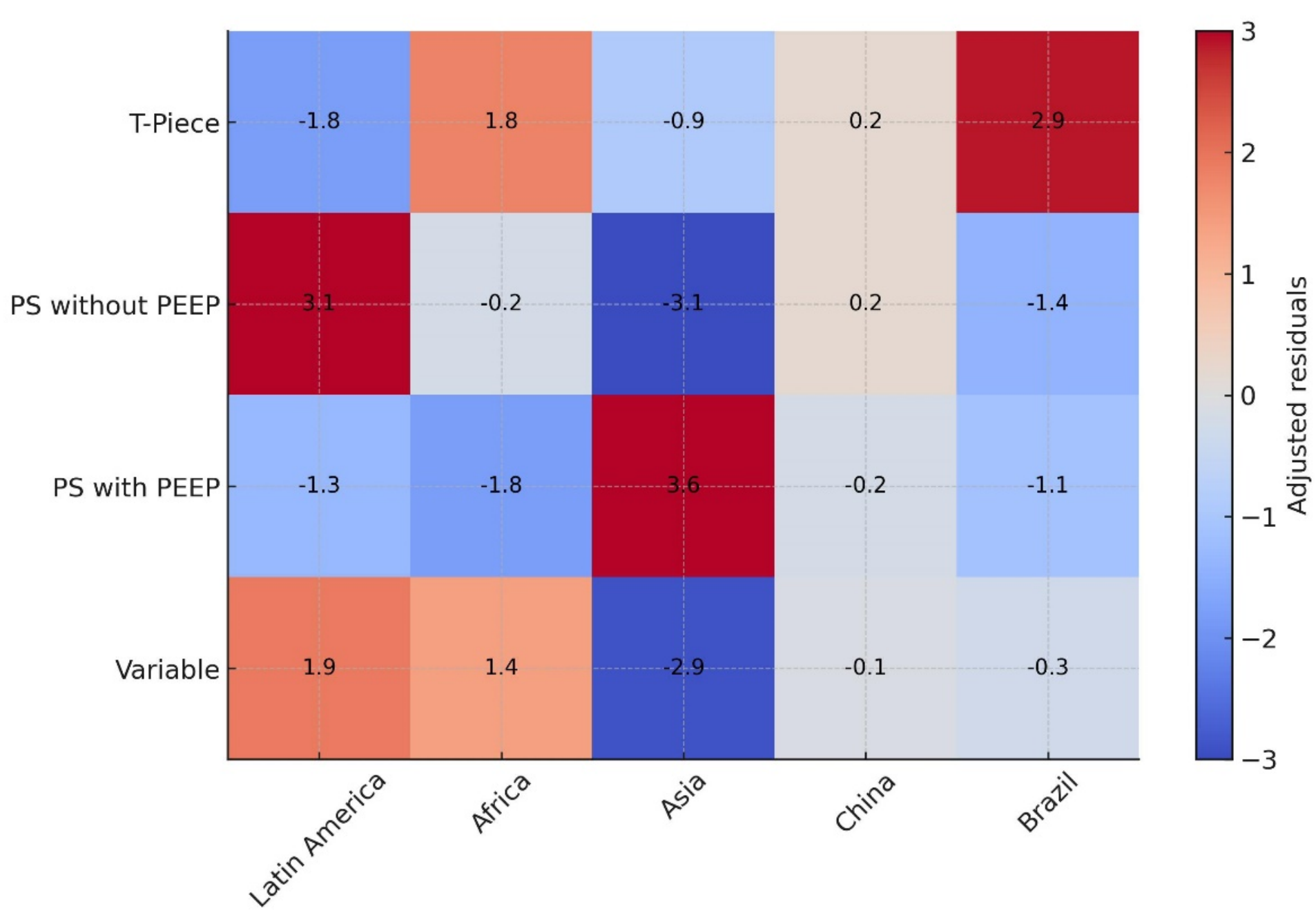


**Figure 2S -** Distribution of the most commonly used spontaneous breathing trial methods across different regions, based on adjusted standardized residuals.

Positive residuals (red) indicate that a given spontaneous breathing trial mode was used more frequently than expected in that region, whereas negative residuals (blue) indicate lower-than-expected use.

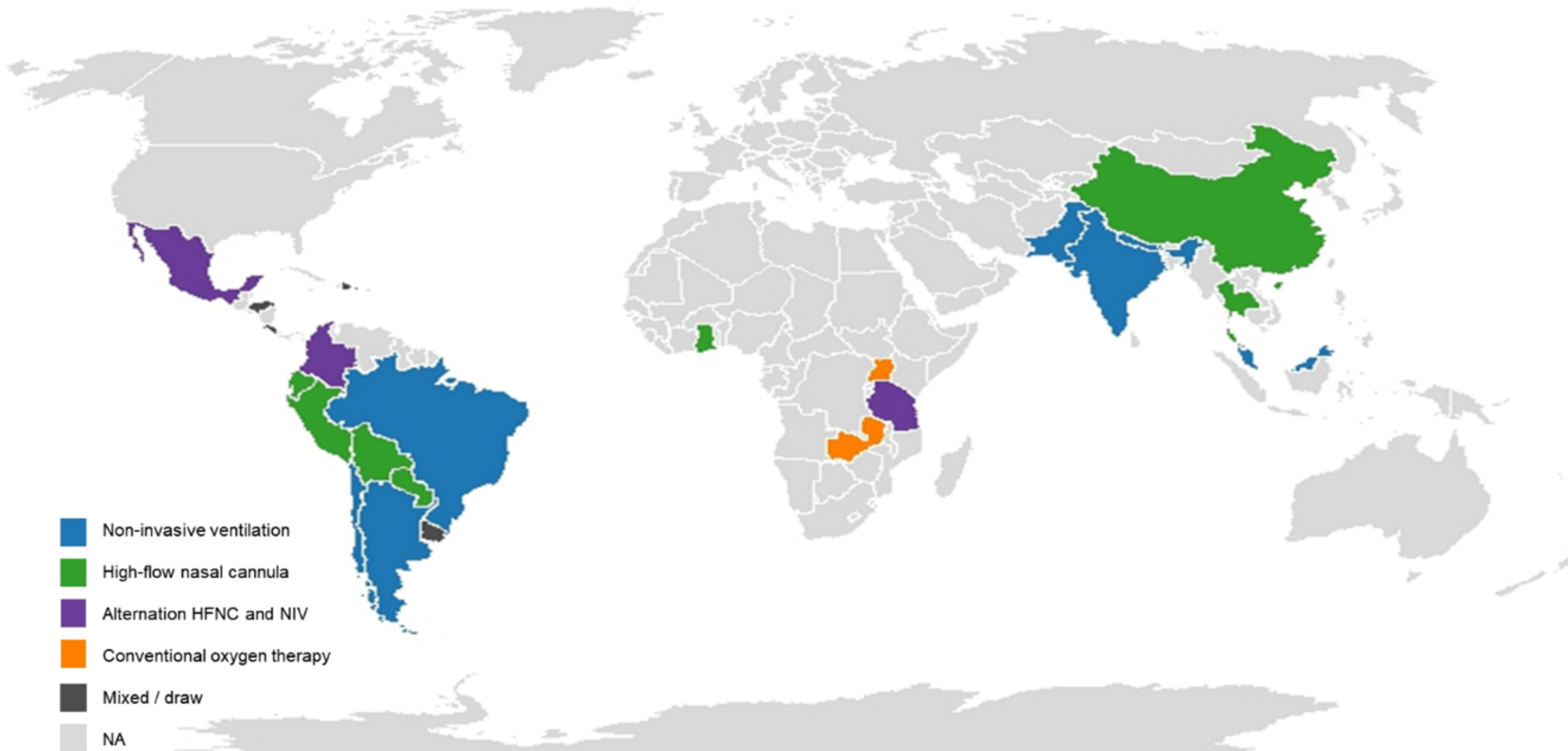


HFNC - high flow nasal cannula; NIV - non-invasive ventilation; NA - not available.

**Figure 3S -** Most frequent post-extubation therapy per country in patients with high risk of extubation failure.

World map illustrating the proportion of responses by country: Brazil 100/115 (87%), China 64/107 (60%), Argentina 22/42 (52.3%), Bolivia 1/1 (100%), Chile 36/58 (62%), Colombia 10/21 (47.6%), Costa Rica 2/2 (100%), Ecuador 14/30 (46.6%), Honduras 1/1 (100%), Mexico 46/89 (51.6%), Paraguay 13/30 (43.3%), Peru 10/21 (47.6%), Uruguay 9/16 (56.2%), Ghana 12/20 (60%), Tanzania 2/3 (66.6%), Uganda 4/9 (44.4%), Zambia 1/1 (100%), India 20/35 (57.1%), Malaysia 25/36 (69.4%), Nepal 24/31 (77.4%), Pakistan 41/66 (62.1%), Thailand 2/3 (66.6%).

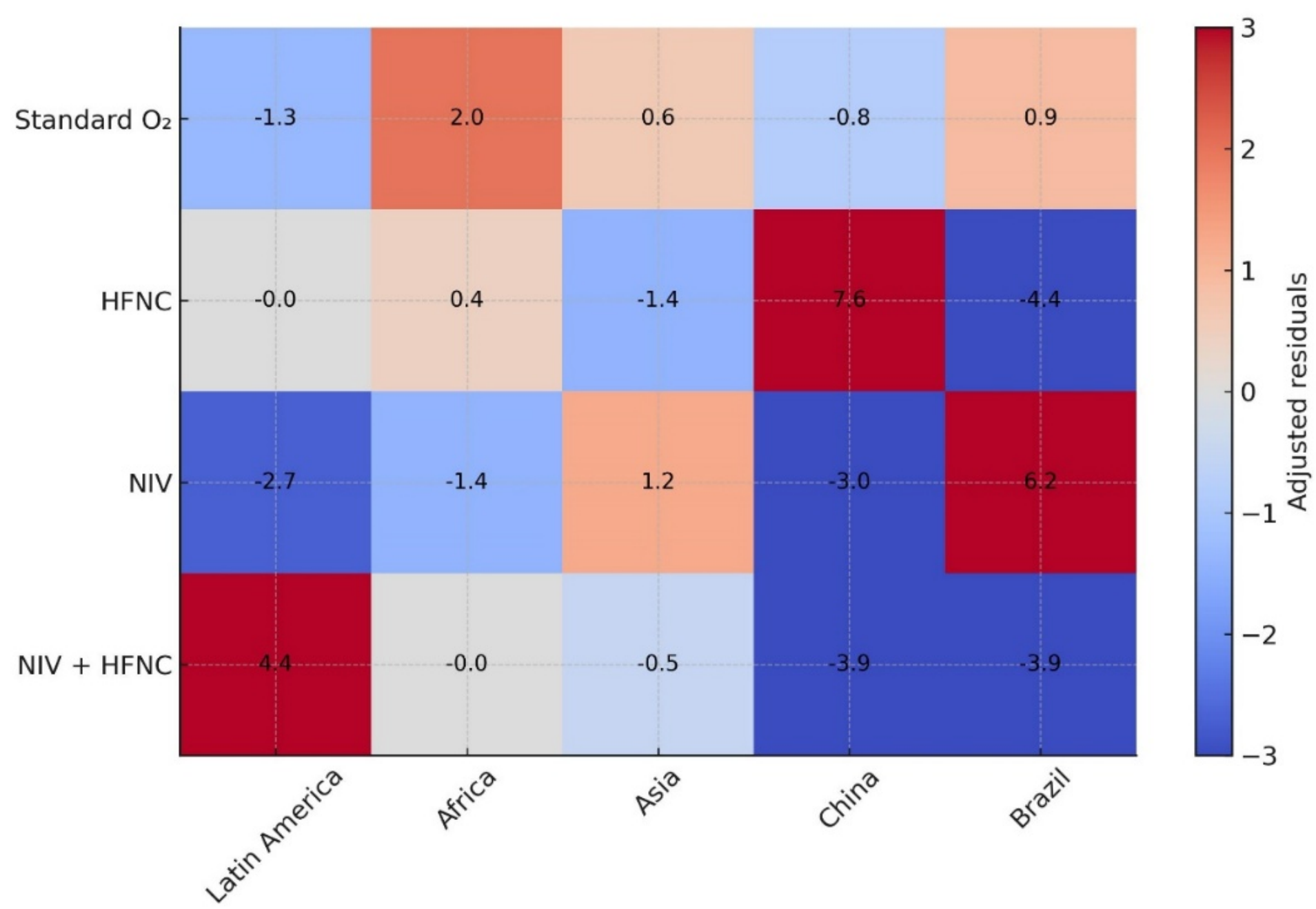


HFNC - high flow nasal cannula; NIV - non-invasive ventilation.

**Figure 4S -** Distribution of the most commonly used post-extubation therapy across different regions, based on adjusted standardized residuals.

Positive residuals (red) indicate that a given post-extubation therapy was used more frequently than expected in that region, whereas negative residuals (blue) indicate lower-than-expected use.

**Table 3S -** Global distribution of participants recruited in the study

| Country | n (%) |
|---|---|
| Latin America | |
| Brazil | 115 (15.6) |
| Argentina | 42 (5.7) |
| Bolivia | 1 (0.1) |
| Chile | 58 (7.8) |
| Colombia | 21 (2.8) |
| Costa Rica | 2 (0.2) |
| Dominican Republic | 1 (0.1) |
| Ecuador | 30 (4.0) |
| Honduras | 1 (0.1) |
| Mexico | 89 (12.0) |
| Paraguay | 30 (4.0) |
| Peru | 21 (2.8) |
| Uruguay | 16 (2.1) |
| Africa | |
| Ghana | 20 (2.7) |
| Tanzania | 3 (0.4) |
| Uganda | 9 (1.2) |
| Zambia | 1 (0.1) |
| Asia | |
| China | 107 (14.5) |
| India | 35 (4.7) |
| Malaysia | 36 (4.8) |
| Nepal | 31 (4.2) |
| Pakistan | 66 (8.9) |
| Thailand | 3 (0.4) |

Total: 738.

**Table 4S -** Strategies and practices to assess extubation readiness

| | Overall (n = 738) | Latin America without Brazil (n = 312) | Asia without China (n = 171) | Africa (n = 33) | Brazil (n = 115) | China (n = 107) |
|---|---|---|---|---|---|---|
| ICUs with a weaning protocol | 541/729 (74.2) (0.70 - 0.77) | 194/309 (62.7) (0.57 - 0.67) | 133/167 (79.6) (0.72 - 0.85) | 21/33 (63.6) (0.46 - 0.77) | 91/113 (80.5) (0.72 - 0.86) | 102/107 (95.3) (0.89 - 0.97) |
| Who leads weaning* | | | | | | |
| Respiratory therapist or physiotherapist | 224/738 (30.4) (0.27 - 0.33) | 94/312 (30.1) (0.25 - 0.35) | 48/171 (28.0) (0.21 - 0.35) | 2/33 (6.0) (0.01 - 0.19) | 60/115 (52.1) (0.43 - 0.61) | 20/107 (18.6) (0.12 - 0.27) |
| Nurse | 24/738 (3.3) (0.02 - 0.04) | 2/312 (0.6) (0.001 - 0.02) | 20/171 (11.6) (0.07 - 0.17) | 1/33 (3.0) (0.005 - 0.15) | 1/115 (0.8) (0.001 - 0.04) | 0/107 (0) (0 - 0.03) |
| Physician | 447/738 (60.6) (0.57 - 0.64) | 178/312 (57.0) (0.51 - 0.62) | 109/171 (63.7) (0.56 - 0.70) | 30/33 (91.0) (0.76 - 0.96) | 37/115 (32.1) (0.24 - 0.41) | 93/107 (87.0) (0.79 - 0.92) |
| Team-based | 119/738 (16.1) (0.13 - 0.18) | 62/312 (19.8) (0.15 - 0.24) | 16/171 (9.3) (0.05 - 0.14) | 2/33 (6.0) (0.01 - 0.19) | 35/115 (30.4) (0.22 - 0.39) | 4/107 (3.7) (0.01 - 0.09) |
| Weaning protocol items* | | | | | | |
| Checklist to assess eligibility for weaning and SBT | 407/738 (55.1) (0.51 - 0.58) | 147/312 (47.1) (0.41 - 0.52) | 103/171 (60.2) (0.52 - 0.67) | 15/33 (45.4) (0.29 - 0.62) | 76/115 (66.0) (0.57 - 0.74) | 66/107 (61.6) (0.52 - 0.70) |
| Spontaneous breathing trials | 459/738 (62.2) (0.58 - 0.65) | 165/312 (52.8) (0.47 - 0.58) | 107/171 (62.5) (0.55 - 0.69) | 19/33 (57.5) (0.40 - 0.72) | 75/115 (65.2) (0.56 - 0.73) | 93/107 (87.0) (0.79 - 0.92) |
| Evaluation and management of laryngeal edema risk | 332/738 (45.0) (0.41 - 0.48) | 128/312 (41.0) (0.35 - 0.46) | 72/171 (42.1) (0.34 - 0.49) | 12/33 (36.3) (0.22 - 0.53) | 54/115 (46.9) (0.38 - 0.56) | 66/107 (61.6) (0.52 - 0.70) |
| Cough strength assessment | 343/738 (46.5) (0.42 - 0.50) | 128/312 (41.0) (0.35 - 0.46) | 67/171 (39.1) (0.32 - 0.46) | 6/33 (18.1) (0.08 - 0.34) | 57/115 (49.5) (0.40 - 0.58) | 85/107 (79.4) (0.70 - 0.86) |
| Prophylactic use of NIV and/or HFNC in high-risk patients | 398/738 (53.9) (0.50 - 0.57) | 151/312 (48.3) (0.42 - 0.53) | 86/171 (50.2) (0.42 - 0.57) | 17/33 (51.5) (0.35 - 0.67)) | 64/115 (55.6) (0.46 - 0.64) | 80/107 (74.7) (0.65 - 0.82) |
| Other | 20/738 (2.7) (0.01 - 0.04) | 11/312 (3.5) (0.01 - 0.06) | 2/171 (1.1) (0.003 - 0.04) | 0/33 (0) (0 - 0.10) | 1/115 (0.8) (0.001 - 0.04) | 6/107 (5.6) (0.02 - 0.11) |
| Strategy to assess readiness for extubation | | | | | | |
| SBT | 361/722 (50.0) (0.46 - 0.53) | 165/309 (53.3) (0.47 - 0.58) | 82/168 (48.8) (0.41 - 0.56) | 9/32 (28.1) (0.15 - 0.45) | 65/113 (57.5) (0.48 - 0.66) | 40/100 (40.0) (0.30 - 0.49) |
| Gradual reduction of support without SBT | 32/722 (4.4) (0.03 - 0.06) | 10/309 (3.2) (0.01 - 0.05) | 5/168 (3.0) (0.01 - 0.06) | 3/32 (9.3) (0.03 - 0.24) | 7/113 (6.2) (0.03 - 0.12) | 7/100 (7.0) (0.03 - 0,13) |
| Both | 323/722 (44.7) (0.41 - 0.48) | 133/309 (43.0) (0.37 - 0.48) | 78/168 (46.4) (0.39 - 0.53) | 20/32 (62.5) (0.45 - 0.77) | 39/113 (34.5) (0.26 - 0.43) | 53/100 (53.0) (0.43 - 0.62) |
| Other | 6/722 (0.8) (0.003 - 0.01) | 1/309 (0.3) (0.0005 - 0.01) | 3/168 (1.7) (0.003 - 0.05) | 0/32 (0) (0 - 0.10) | 2/113 (1.8) (0.004 - 0.06) | 0/100 (0) (0 - 0.03) |
| Cough strength assessment | | | | | | |
| Not assessed | 296/696 (42.5) (0.38 - 0.46) | 114/308 (37.0) (0.31 - 0.42) | 94/168 (56.0) (0.48 - 0.63) | 22/32 (68.7) (0.51 - 0.82) | 43/112 (38.3) (0.29 - 0.47) | 23/76 (30.2) (0.21 - 0.41) |
| Peak cough flow meter | 141/696 (20.3) (0.17 - 0.23) | 70/308 (22.7) (0.18 - 0.27) | 11/168 (6.5) (0.03 - 0.11) | 2/32 (6.2) (0.01 - 0.20) | 31/112 (27.6) (0.20 - 0.36) | 27/76 (35.5) (0.25 - 0.46) |
| Internal flowmeter on the mechanical ventilator | 191/696 (27.4) (0.24 - 0.30) | 105/308 (34.0) (0.29 - 0.39) | 29/168 (17.2) (0.12 - 0.23) | 1/32 (3.1) (0.005 - 0.15) | 31/112 (27.6) (0.20 - 0.36) | 25/76 (32.8) (0.23 - 0.44) |
| Other | 66/696 (9.4) (0.07 - 0.11) | 19/308 (6.1) (0.03 - 0.09) | 33/168 (19.6) (0.14 - 0.26) | 7/32 (21.8) (0.11 - 0.38) | 7/112 (6.2) (0.03 - 0.12) | 0/76 (0) (0 - 0.04) |
| Routine cuff leak testing | 607/717 (84.7) (0.81 - 0.87) | 253/308 (82.1) (0.77 - 0.86) | 146/168 (87.0) (0.80 - 0.91) | 24/32 (75.0) (0.57 - 0.86) | 97/113 (85.8) (0.78 - 0.91) | 87/96 (90.6) (0.83 - 0.94) |
| Steroid use in patients at risk of post-extubation laryngeal edema | 576/716 (80.4) (0.77 - 0.83) | 248/309 (80.2) (0.75 - 0.84) | 134/167 (80.2) (0.73 - 0.85) | 28/32 (87.5) (0.71 - 0.95) | 77/112 (68.7) (0.59 - 0.76) | 89/96 (92.7) (0.85 - 0.96) |

ICU - intensive care unit; SBT - spontaneous breathing trial; NIV - non-invasive ventilation; HFNC - high flow nasal cannula. * One or more alternatives could be selected. Data are shown as proportions and percentages, with 95% confidence intervals.
